# The Detection and Morphological Analysis of Circulating Tumor and Host Cells in Breast Cancer Xenograft Models

**DOI:** 10.3390/cells8070683

**Published:** 2019-07-05

**Authors:** Loredana Cleris, Maria Grazia Daidone, Emanuela Fina, Vera Cappelletti

**Affiliations:** Biomarkers Unit, Department of Applied Research and Technological Development, Fondazione IRCCS Istituto Nazionale dei Tumori, 20133 Milan, Italy

**Keywords:** circulating tumor cells, metastasis, xenograft models, breast cancer

## Abstract

Hematogenous dissemination may occur early in breast cancer (BC). Experimental models could clarify mechanisms, but in their development, the heterogeneity of this neoplasia must be considered. Here, we describe circulating tumor cells (CTCs) and the metastatic behavior of several BC cell lines in xenografts. MDA-MB-231, BT-474, MDA-MB-453 and MDA-MB-468 cells were injected at the orthotopic level in immunocompromised mice. CTCs were isolated using a size-based method and identified by cytomorphological criteria. Metastases were detected by COX IV immunohistochemistry. CTCs were detected in 90% of animals in each model. In MDA-MB-231, CTCs were observed after 5 weeks from the injection and step wisely increased at later time points. In animals injected with less aggressive cell lines, the load of single CTCs (mean ± SD CTCs/mL: 1.8 ± 1.3 in BT-474, 122.2 ± 278.5 in MDA-MB-453, 3.4 ± 2.5 in MDA-MB-468) and the frequency of CTC clusters (overall 38%) were lower compared to MDA-MB-231 (946.9 ± 2882.1; 73%). All models had lung metastases, MDA-MB-453 and MDA-MB-468 had ovarian foci too, whereas lymph nodal involvement was observed in MDA-MB-231 and MDA-MB-468 only. Interestingly, CTCs showed morphological heterogeneity and were rarely associated to host cells. Orthotopic xenograft of BC cell lines offers valid models of hematogenous dissemination and a possible experimental setting to study CTC-blood microenvironment interactions.

## 1. Introduction

Metastasis is definitely a hallmark of cancer [1] and represents the main cause of cancer-related deaths [2] due to ineffective therapies. Unraveling the molecular mechanisms of tumor progression would help to anticipate disease outcome and to point the way for selecting personalized treatments. In breast cancer (BC), in particular, the timing of cancer cell dissemination has been largely discussed [3] and has proven to represent an early step in tumor progression [4,5]. In accordance with this, circulating tumor cells (CTCs) can be detected in patients without clinical evidence of secondary lesions [6,7,8] and, in several studies, the presence of dormant cells has been also reported even in the bone marrow of patients with ductal carcinoma in situ [9,10,11]. In addition to this grim scenario, BC is, in fact, a group of heterogeneous tumors [12,13,14,15], with cancer cells cross-talking with normal cells from the microenvironment [16,17]. More recently, based on copy number and gene expression data from over 2000 tumors, BCs were re-classified into ten clusters associated with distinct clinical outcomes [18,19], with implications for patient management. As the development of drug resistance is often interpreted as an inevitable consequence of tumor heterogeneity [20,21], efforts to address such interrelated themes are urgently needed, especially in non-operable and advanced-stage clinical settings.

At present, the biological events and molecular mechanisms that orchestrate the metastatic process are still not fully understood due to their complexity [22,23,24]. Functional assays to elucidate the biological meaning of a gene in tumor dissemination or the effect of a compound on metastasis outgrowth have to be necessarily set in organisms. In this field, scientists have largely based their studies on metastasis modeling on laboratory animals, including drosophila, zebrafish, mice, rats and, more rarely, rabbits, companion pets and monkeys with spontaneous onset of cancer [25]. Xenotransplantation of BC cell lines in mice with a compromised immune system is commonly used as a model for metastasis studies. In particular, direct injection into the systemic circulation of the MDA-MB-231 cell line and its derivatives generated several models of metastasis [26,27,28,29,30], either in basal conditions or after selection of organ-specific metastatic variants upon several rounds of transplantation [31,32], providing valuable knowledge on the genetic determinants of metastasis in BC. However, although forced hematogenous dissemination does enable to finely dissect the late steps of the metastatic cascade [31], this strategy is not adequate to recapitulate the initial events of the process as in spontaneous metastasis models, where cells are implanted at the orthotopic level. Moreover, the research mainly focused on a single model type might fail in addressing the heterogeneity issue in BC, thus limiting possible applications to the clinical context [33].

Since the molecular classification of BC has been established, researchers have paid attention to the similarities between cell lines and clinical samples. Studies have shown that the luminal, basal, HER2 and claudin-low clusters identified in BC are mirrored in BC cell lines [34,35,36]. However, the claudin-low and basal subtypes are over-represented among the BC cell lines used for xenograft models [37]. Indeed, spontaneous metastasis is a rare event when using cell lines belonging to less aggressive subtypes, and only a few models with variable frequencies of metastasis have been described in recent years for MCF7, BT-474 and MDA-MB-453 [38,39]. Not dissimilar from BC cell lines, which however ensure a high tumor take in mice, is the behavior of xenotransplanted BC specimens (PDXs, patient-derived xenografts), whose both development and metastatic organotropism in mice are variable and dependent on the aggressiveness of the tumor of origin. Indeed, despite PDXs representing important preclinical tools since proven to retain over serial passages histopathology, behavior and genomic features of the tumor of origin [40,41,42,43,44,45], in BC the tumor take efficiency of the luminal subtype in mice is low [46,47], thus generating a bias towards aggressive triple-negative BCs models.

On the basis of these considerations, we have reconsidered the use of xenograft models from BC cell lines for basic metastasis research studies. To this aim, we have (i) transplanted BC cell lines belonging to different molecular subtypes in the mammary fat pad of immunocompromised mice, (ii) set up a method to detect CTCs and small foci of metastatic cells in such xenograft models, and (iii) described the morphological features of BC cell line derived CTCs and host-derived circulating cells.

## 2. Materials and Methods

### 2.1. Cell Lines

Cell lines were purchased from the American Type Culture Collection organization and verified for identity via short tandem repeat (STR) profile analysis using the StemElite™ ID System kit (Promega, Madison, WI, USA), which yielded a 100% match on 9 STR loci, and on amelogenin for gender identification, in all cases.

BT-474, MDA-MB-453 and MDA-MB-468 BC cell lines were cultured in Dulbecco’s Modified Eagles’ Medium (DMEM)/F-12 medium (Lonza, Switzerland) supplemented with 10% South America Fetal Bovine Serum (FBS, Lonza). The MDA-MB-231 BC cell line was cultured in DMEM/F-12 medium supplemented with 5% FBS. Cells were grown at 37 °C in a 95% humidified 5% pCO_2_ atmosphere.

All experiments were performed using cells from the second to the eighth in vitro passage from thawing, and showing at least 95% viability by 0.4% Trypan Blue solution exclusion test. Cell culture supernatants were regularly tested for Mycoplasma contamination using the MycoAlert^TM^ Mycoplasma Detection Kit (Lonza) before each injection in mice.

### 2.2. Animal Models

Animal experiments were performed according to the Italian law D.L. 116/92, and the following additions, which enforced the 2010/63/EU Directive. The study protocols were approved by the Ethical Committee for Animal Experimentation at Fondazione IRCCS Istituto Nazionale dei Tumori (INT), in Milan, (INT_08/2012, and INT_01/2017, which was also approved by Italian Ministry of Health with approval number 452/2017-PR, following the receipt of the D.L. 26/2014). All efforts were deployed to minimize animal suffering [48], following the most recently published version of recommended ARRIVE guidelines [49]. Female NOD.CB17-*Prkdc^scid^*/J (NOD scid) and NOD.*Cg*-*Prkdc^scid^ Il2rg^tm1Wjl^*/SzJ (NSG) mice were purchased from Charles River (Wilmington, MA, USA) and The Jackson Laboratory (Sacramento, CA, USA), respectively, and bred by the qualified personnel at INT Animal House Facility in individually ventilated cages, 3 to 5 animals per cage. Animals were anesthetized by intraperitoneal injection of a ketamine (100 mg/kg) and xylazine (5 mg/kg) cocktail before orthotopic injection of cancer cells and before animal sacrifice. Sacrifice procedure was cervical dislocation, performed at a priori set experimental time points or immediately upon signs of moderate suffering (e.g., decrease in activity, hunched appearance, ruffled hair coat, respiratory distress).

The tumor implant was performed under sterile conditions on healthy and normal-weight 7- to 16-week-old anesthetized mice using a 30G needle syringe. Eighty to ninety µL of Dulbecco’s Phosphate Buffered Saline (DPBS, Lonza) cell suspensions mixed with 50% ECM Gel from Engelbreth-Holm-Swarm murine sarcoma (Sigma-Aldrich, St. Louis, MO, USA) Matrigel matrix (final concentration 4 mg/mL) were injected in the mammary fat pad (m.f.p.) of the axillary and/or the inguinal mammary gland, according to the scheme reported in Table 1.

BT-474 cell injection was performed after 24–48 h from subcutaneous implantation of a 0.72 mg 90-day release 17-β-estradiol pellet (Innovative Research of America, Sarasota, FL, USA), performed on the neck lateral side using a trocar. The overall tumor take rate was 100%.

For the time-course experiments with MDA-MB-231 cells, 4 groups of 6 animals each were injected with cells according to the standard scheme. Animals were randomized before sacrifice at the defined time points (day 35, 50, 65 and 80) according to the tumor growth rate and the cage where they had been bred. Tumor take was obtained in 23/24 mice.

Tumor growth was monitored every week using a caliper and the tumor mass (g) was estimated by the (*D* × *d^2^*)/2 formula, where *D* and *d* represent the longest and the shortest diameter, respectively, of the nodule. The tumor load was lower than 10% of the body mass (range: 0.4–9.5%), except for two animals (10.2% and 15.7%) in which tumors had increased rapidly during the latest week.

An intravenous injection was performed using suspensions of 10^6^ or 2 × 10^6^ cells in 400 μL of DPBS.

Splenic leukocytes from BALB/c Nude mice were kindly provided by Dr. Claudia Chiodoni from the Molecular Immunology Unit at INT.

### 2.3. Collection of Tissues and Organs

Blood samples were drawn from anesthetized mice by cardiac puncture, using a 1 mL 26G needle EDTA conditioned syringe (1.8 mg/mL final concentration), stored at 4 °C and processed for CTC isolation within 30 min. Mice were immediately sacrificed and primary tumor nodules and organs (lung, axillary, inguinal subclavian or peritoneal lymph-nodes, ovaries, liver, kidneys, brain, and spleen) were collected and fixed in a 10% neutral buffered formalin solution (Bio-Optica, Milan, Italy) for 18–24 h; samples were then washed with distilled water and stored in 70% ethanol until paraffin embedding.

### 2.4. Circulating Tumor Cell Isolation and Detection

CTCs were isolated using the ScreenCell® Cyto kit (ScreenCell, Sarcelles, France), according to the manufacturer instructions. Briefly, blood was diluted in DPBS to reach 3 mL and subsequently mixed with 4 mL of the ScreenCell® FC2 proprietary buffer for red blood cell osmotic lysis and cell fixation. When the flux rate decreased due to a microcoagulation phenomenon or the presence of numerous CTCs, the residual blood was filtered on further devices. After filtration, the isolation supports (IS) were stained with Hematoxylin Solution S (Merck, Darmstadt, Germany) for 1 min and a Shandon Eosin Y Aqueous Solution (Thermo Fisher Scientific Inc., Waltham, MA, USA) for 30 s, or with a pure May-Grünwald solution for 2.5 min, followed by a 2.5-min incubation step with a May-Grünwald solution diluted 1:2 with pH 7-adjusted distilled water, and a 10-min incubation step with a Giemsa solution (Merck) 1:10 diluted with pH 7-adjusted distilled water. All samples were analyzed by a referral pathologist at ScreenCell. The cytomorphological analysis and CTC count were performed on the basis of the criteria of malignancy reported by Hofman et al. [50]. Major criteria for CTC identification were a high nucleus-to-cell ratio (i.e., cytoplasm area/whole cell area, ≥0.5) and large nuclear size (≥20 μm diameter), whereas minor criteria included irregular nuclear contours and nuclear hyperchromatism. CTC clusters were defined as groups of two or more CTCs, sometimes mixed with platelets and various leukocytes (i.e., circulating tumor microemboli, CTM), showing criteria of malignancy like those described for single CTCs. The nucleus-to-cell ratios in CTC aggregates are similar to those in single CTCs in [51]. Platelets appear as small, round eosinophilic or grayish particles, and can be found isolated or grouped in plaques, sometimes mixed with deposits of fibrin. Like CTCs, lymphocytes have a high nucleus-to-cell ratio, but they are smaller (7–8 µm diameter). Circulating atypical giant cells were defined as large cells (20–300 μm diameter), with generally voluminous and filamentary cytoplasm, various morphology (e.g., amorphous, round, elongated) and nucleus to cell ratio lower than that of CTCs [52,53]. Samples were defined as CTC-positive (+ve) when at least one single CTC and/or CTC cluster and/or CTM were observed in at least one stained IS.

### 2.5. Immunofluorescence and Immunohistochemical Staining

Immunofluorescence was performed on unstained ISs upon storage at −20 °C. ISs were incubated in an oven at 37 °C for 1 h, rehydrated in Tris Buffered Saline (TBS) 1× pH 7.4 (Bio-Optica) and blocked for 30 min with 5% bovine serum albumin (BSA, Sigma-Aldrich, St. Louis, MO, USA) in TBS 1X. Tumor cells were stained overnight at 4 °C using a rabbit monoclonal Alexa Fluor® 488 conjugated antibody against human cytochrome *c* oxidase subunit IV (COX IV, clone 3E11, isotype IgG; Cell Signaling Technology, Danvers, MA, USA), diluted 1:100 in 5% BSA in TBS 1×. Nuclei were stained with a 5 µg/mL 4′,6-Diamidino-2-phenylindole (DAPI) dilactate solution (Sigma-Aldrich, St. Louis, MO, USA). ISs were mounted on glass slides and covered with a round coverslip using the Fluoroshield Mounting Medium (Abcam, Cambridge, UK). Images were acquired by Nikon Eclipse TE2000-S fluorescence (Nikon, Tokyo, Japan) microscope.

Four-micron thick formalin-fixed paraffin-embedded (FFPE) sections from tumor nodules and organs were deparaffinized by standard protocols and stained using a rabbit monoclonal antibody against human COX IV (clone 3E11, isotype IgG, Cell Signaling Technology, Danvers, MA, USA). Antigen retrieval was performed at 95 °C for 30 min in a Sodium Citrate Buffer (10 mM Sodium Citrate, 0.05% Tween 20, pH 6.0). Endogenous biotin blocking was performed for liver sections only using the Dako Cytomation Biotin Blocking System (Dako, Troy, MI, USA). Samples were incubated with a 1:1000 diluted (Antibody Diluent, Dako) primary antibody at 4 °C overnight. Antibody visualization was obtained using the EnVision®+ System-HRP Labelled Polymer (Dako). Nuclei were counterstained with a Mayer’s Hematoxylin Solution (Bio-Optica). Sections were observed and images acquired by a Nikon Eclipse E600 microscope.

For COX IV specificity verification, 4 consecutive sections from different organs of 3 non-tumor-bearing NOD scid mice were analyzed. For the MDA-MB-231 time-course experiment, 4 sections per lymph-node and 10 sections per lung sample were analyzed. Macroscopic inspection of organs at sacrifice and microscopic analysis by IHC on a series of non-adjacent FFPE sections (series of 4 consecutive stained and 8 consecutive unstained sections), for a total of 24 or 48, according to positivity, were performed for the preliminary assessment of metastasis formation in all kind of models (Experiment 1). For the quantitation of metastasis-positive (+ve) sections (Experiment 2), systematic IHC analysis was focused on a series of 24 or 48 non-adjacent FFPE sections (a series of 8 consecutive stained and 8 consecutive unstained sections) from lung, lymph-nodes and ovary samples. For the artificial metastasis experiment by tail-vein injection, 4 FFPE consecutive sections per lung sample were analyzed.

### 2.6. Statistical Analysis

Statistical analyses and graph constructions were performed using Graph Pad Prism v5. Differences in tumor mass between axillary and inguinal nodules were assessed using the point by point multiple Student’s *t*-test, assuming that all time points were samples from populations with the same standard deviation, and the false discovery rate was set at 1% and determined using the two-stage linear step-up procedure of Benjamini, Krieger and Yekutieli [54].

## 3. Results

### 3.1. Technical Protocol for CTC Isolation and Species-Specificity-Based Detection of Tumor Cells in Xenograft Models

**Figure 1:**
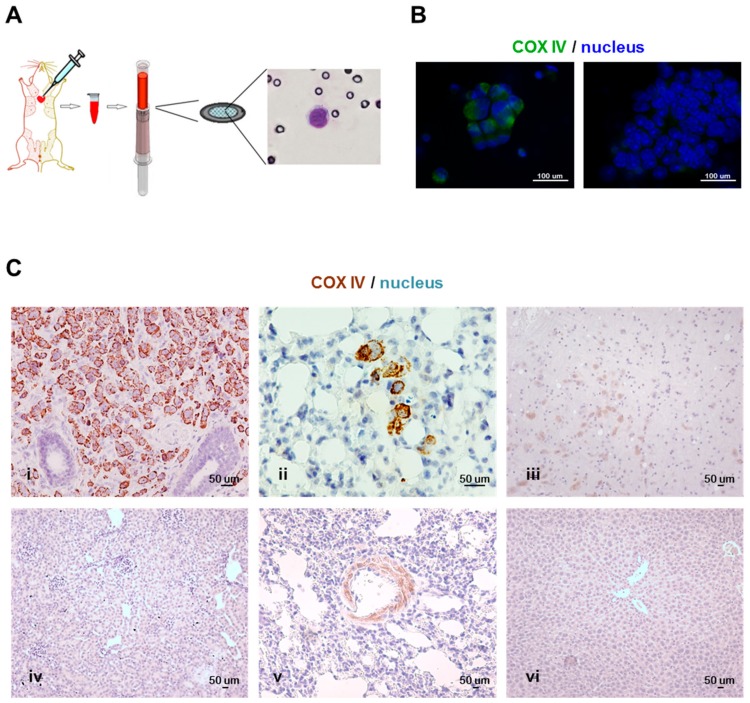
The methodology for the detection of circulating tumor cells (CTCs) and metastases in xenograft models. (**A**) The scheme illustrates a CTC isolation and detection technical approach for application in xenograft mouse models. Briefly, blood was drawn by cardiac puncture and cells were isolated by filtration on a porous membrane and identified by the morphological criteria (or immunostaining). Images represent (**B**) a cluster of CTCs (left) and a cluster of leukocytes (right) isolated from MDA-MB-231 xenografts, acquired by the 4′,6-Diamidino-2-phenylindole (DAPI) and the Fluorescein isothiocyanate (FITC) filters (60× oil immersion objective) and showing COX IV positive and negative staining, respectively; (**C**) COX IV immunohistochemistry stained formalin-fixed paraffin-embedded sections of (**i**) primary tumor nodule (20× objective) and (**ii**) lung metastases (40× objective) from MDA-MB-231 xenograft, and of (**iii**) brain, (**iv**) kidney, (**v**) lung, and (**vi**) liver (10× objective) collected from a non-tumor-bearing mouse.

For CTC isolation, blood samples were drawn from anesthetized mice by cardiac puncture, which was proven to ensure the highest CTC yield compared to other approaches, according to Eliane et al. [55], and processed with the size-based CTC isolation device provided by ScreenCell® (Figure 1, Panel A; details are reported in Materials and Methods).

CTCs were identified on the basis of the cytomorphological criteria of malignancy already described for cancer patients [50]: in xenograft models, CTCs showed (i) a larger nucleus (generally 13 to 15 μm in diameter) compared to leukocytes, whose nuclei instead appeared slightly larger (about 7–8 μm in diameter) than membrane pores (6.5 ± 0.33 μm), (ii) a high nucleus-to-cell ratio (>0.5 for cell lines, rather than 0.75, cut-off used for clinical samples), (iii) a dense basophilic and irregularly outlined nucleus and (iv) a pale-bluish ring of cytoplasm, which generally appears as a thin rim encircling the nucleus.

Such a blood sampling approach coupled with filtration showed high efficiency in terms of sensitivity, as described in the following paragraphs, and adaptability to murine blood sample processing and cytological analysis for CTC detection, since the sample quality in terms of cellularity was adequate and the cell morphology was well preserved in about 80% (58/71) of samples.

Furthermore, a technical protocol was developed to effectively isolate and unambiguously identify tumor cells in tissues from xenograft mouse models, taking advantage of the human-murine species-specificity.

Given the weak metastatic ability expected in some models, an antibody-based staining protocol was set up in order to facilitate both the quantitation of rare CTCs and to enable screening for metastases in FFPE tissue sections from samples with microscopic and scattered metastatic foci. Immunofluorescence (for CTCs) and immunohistochemistry (IHC) analyses (for tissue sections) were performed using a commercially available antibody specific for the human mitochondrial marker cytochrome *c* oxidase subunit IV (COX IV). The non-cross-reactivity of the antibody with the murine counterpart has been preliminarily verified by immunofluorescence on peripheral blood mononuclear cells from a BALB/c nude mouse used as the control (Figure S1), and by IHC on FFPE sections of several organs from non-tumor-bearing NOD scid mice (Figure 1, Panel C), thus proving to be a reliable method to detect tumor cells in mouse xenografts.

CTCs were detectable by immunofluorescence and distinguished from leukocytes by the nucleus size and the typical staining pattern, as depicted in Figure 1, Panel B: CTCs organized in clusters have intense cytoplasmic-specific staining and are larger compared to the cluster of leukocytes, which instead have smaller nuclei and show negative staining for COX IV.

### 3.2. Cancer Cell Dissemination Can Be Monitored from the Early to Late Stages of Tumor Progression in the MDA-MB-231 Xenograft Model

**Figure 2:**
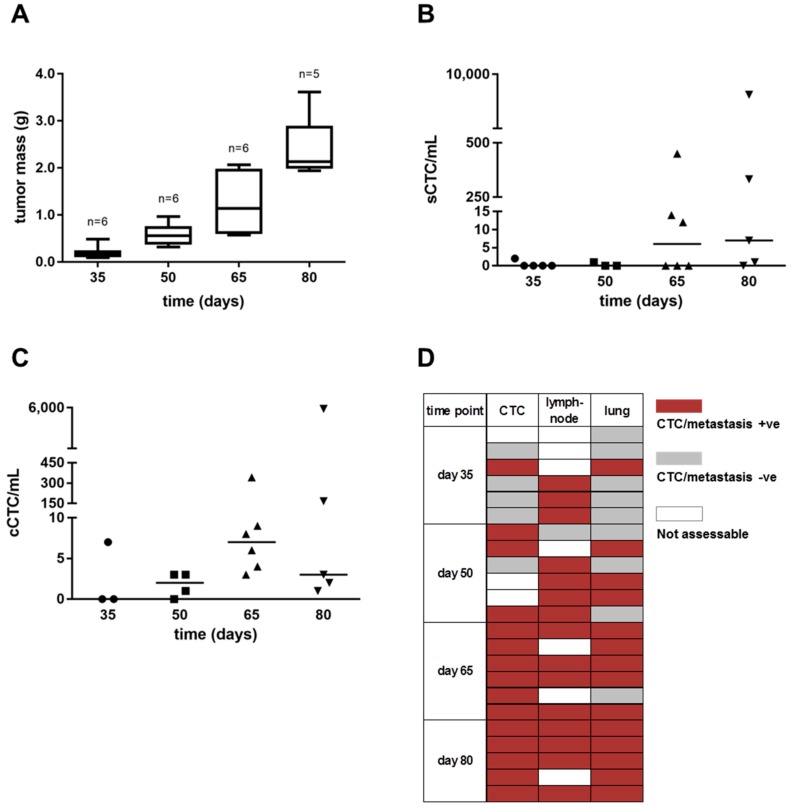
The detection of CTCs and metastases at the early and late stages of tumor progression in the MDA-MB-231 xenograft model. Box and whiskers plots and dot plots represent the distribution of (**A**) the total tumor mass, (**B**) single CTC (sCTC) and (**C**) CTC cluster or tumor microemboli (cCTC) numbers per milliliter of blood (horizontal line representing the median value), at different experimental time points. (**D**) the scheme represents the frequency of CTC-positive (+ve) and of lymph-nodal or pulmonary metastasis-positive (+ve) animals per group, at each experimental time point.

The dynamics of dissemination in the MDA-MB-231 model was investigated in a time-course experiment, where the CTC load and the frequency of lymph-nodal and pulmonary metastases were measured at different time points after tumor cell injection. Overall, the load of single CTCs (mean ± SD: 0.40 ± 0.89; 0.33 ± 0.58; 79.33 ± 181.7; 1,993 ± 4,269; Figure 2, Panel B) and CTC clusters (mean ± SD: 2.33 ± 4.04; 1.75 ± 1.50; 62.00 ± 137.20; 1,229 ± 2,653; Figure 2, Panel C) showed a stepwise increase during progression, which mirrored the primary tumor growth (Figure 2, Panel A). Following a similar trend, the frequency of metastasis +ve cases, assessed in lymph-nodes (axillary, inguinal, subclavian or peritoneal) and lungs, increased during time, although, differently than lungs, metastases at lymph-nodes were detectable since the earliest phases from tumor injection (Figure 2, Panel D). At day 35 CTCs were found in 1 out of 5 assessable cases (2 CTCs, Figure 2, Panel B), consistently with the detection of few metastatic cells at lung in the same animal (Figure 2, Panel D). At day 50 lung metastases were found in 1 out of 3 CTC +ve cases only. On the contrary, 5 out of 6 cases at day 65 and 5 out of 5 cases at day 80 had both CTCs and pulmonary metastases.

MDA-MB-231 cells were also injected in the tail vein of five animals and their presence in blood was monitored during time. Blood samples collected from two animals, injected with 10^6^ or 2 × 10^6^ cells, 1 h after injection contained 1 and 7 sCTCs per milliliter, respectively, thus indicating that the vast majority of cells had reached peripheral districts in short time from forced blood dissemination. The remaining three animals, two injected with 10^6^ and one injected with 2 × 10^6^ cells, were sacrificed after 78 days and were all CTC +ve and lung metastasis +ve. cCTCs were detected in all cases and ranged from 1 to 31 per milliliter, while sCTCs (about 280) were found in one animal only, injected with 10^6^ cells. Lymph-nodes, ovaries and spleen were all negative for metastases by macroscopic examination and IHC analysis.

### 3.3. Breast Cancer Cell Lines with Different Subtypes Disseminate in Blood and Show Distinct Organotropism in Xenograft Models

**Figure 3:**
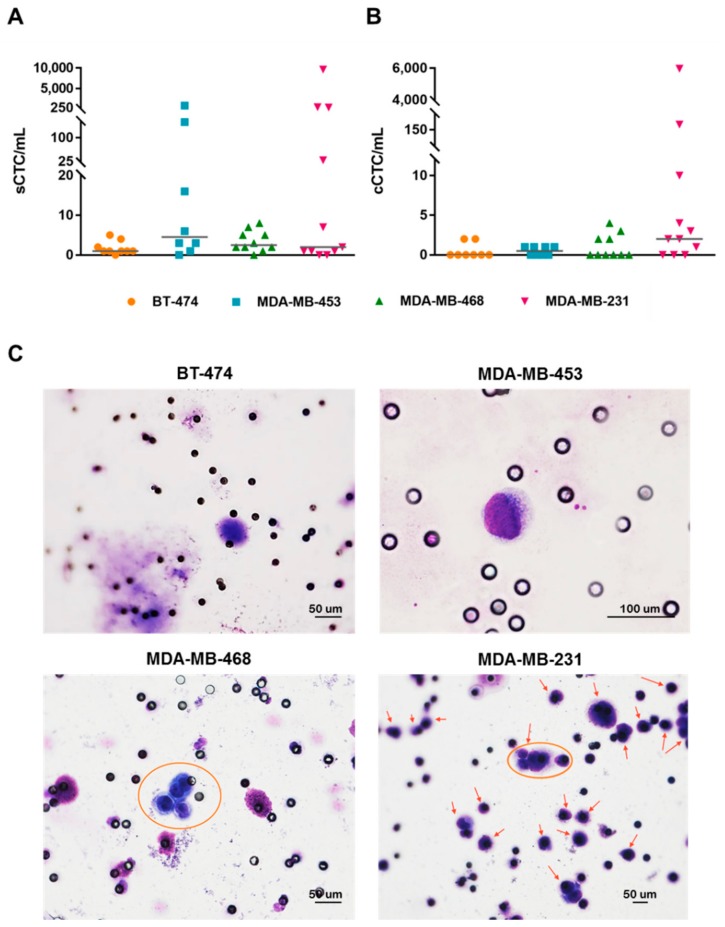
The single CTC (sCTC) and CTC cluster (cCTC) numbers in breast cancer xenograft models. Dot plots represent the distribution (horizontal line corresponding to the median value) of (**A**) sCTCs and (**B**) cCTCs per milliliter of blood. Images (**C**) represent May-Grünwald-Giemsa stained sCTC (40× objective) from BT-474, sCTC (60× oil immersion objective) from MDA-MB-453, cCTC consisting of four cells from MDA-MB-468 (circle, 40×), numerous sCTCs and a cCTC consisting of 4-to-5 cells (arrows, 20× objective) from MDA-MB-231 xenografts.

CTC models were obtained by the orthotopic injection of BT-474, MDA-MB-453, MDA-MB-468 and MDA-MB-231 BC cell lines, performed in two independent experiments. The tumor take rate was 100% in all models and the growth rate of nodules was faster in MDA-MB-231 xenografts, where the total mass reached 500 mg after about 50 days from the cell injection, compared to the other models, which reached comparable masses over longer times (Figure S2), thus mirroring the expected level of aggressiveness according to the molecular subtype of each cell line. A significant difference (adjusted p-value <0.01) between the tumor mass of axillary and inguinal nodules was also observed in MDA-MB-468 (axillary versus inguinal mean ± SD tumor mass (g): 0.89 ± 0.22 versus 0.62 ± 0.27 and 1.00 ± 0.31 versus 0.69 ± 0.30, after 92 and 98 days from tumor implant, respectively) and MDA-MB-231 xenografts (axillary versus inguinal mean ± SD tumor mass (g): 1.02 ± 0.38 versus 0.69 ± 0.27, 1.12 ± 0.40 versus 0.77 ± 0.21, and 1.53 ± 0.64 versus 0.83 ± 0.27, after 75, 78, and 83 days from the tumor implant, respectively), with a general trend towards a faster growth rate in the axillary compared to the inguinal mammary fat pad injection site (Figure S2). Interestingly, MDA-MB-231 and the less aggressive BC cell lines were both able to disseminate in blood as sCTCs, (Figure 3, Panel A) found in about 90% of cases, and as cCTCs (both tumor cell clusters and microemboli, Figure 3, Panel B), detected at variable frequency according to the specific xenograft model. Overall, in both experiments, the sCTC and cCTC load per milliliter of blood was higher in the MDA-MB-231 (median(range): 2(0–9625) and 2(0–5973), respectively) compared to the other CTC models (Table S1), in keeping with the aggressiveness and high proliferation rate of these cells.

Among the weakly metastagenic models, MDA-MB-453 showed the highest numbers of sCTC/mL (median(range): 4.5(0–800)), while overall sCTC numbers for BT-474 and MDA-MB-468 ranged from 0 to 8. Moreover, cCTC positivity was approximately 2-fold lower in the less aggressive models (overall 10/26 cases) compared to the highly metastatic MDA-MB-231 xenografts (8/11 cases). Representative images of sCTCs and cCTCs from each model are reported in Figure 3, Panel C.

The metastatic potential of BC cell lines was also assessed in a preliminary exploratory experiment by macroscopic inspection and IHC analysis. Overall, organs presenting with metastasis were lung, lymph-nodes and ovaries, and those without metastasis +ve sections were the liver, brain and spleen. In a second experiment, systematic IHC analysis (Table 2) confirmed that ovarian metastases were detectable in 2 out of 7 MDA-MB-468 and the majority of MDA-MB-453 xenografts, but not in the BT-474, and likely MDA-MB-231 models, since they did not show ovary enlargement at macroscopic inspection. Lymph-nodal involvement was already macroscopically assessable in the MDA-MB-468 and MDA-MB-231 models in 100% of cases, as also confirmed by the IHC analysis, whereas lymph-nodes in BT-474 and MDA-MB-453 models were hardly detectable and collectible, suggesting the absence of massive dissemination via the lymphatic system. Instead, lungs were the metastatic site showing the highest tropism and frequency in all xenograft models. Consistently with CTC numbers, metastatic foci in weakly aggressive models consisted of single scattered cells or small foci of 3–30 cells each compared to the larger clusters, and macroscopic nodules in a few cases, observed in MDA-MB-231 xenografts (Figure S3).

### 3.4. Circulating Tumor Cells in Breast Cancer Xenograft Models are Pleomorphic and Circulate with Cells of the Host

**Figure 4:**
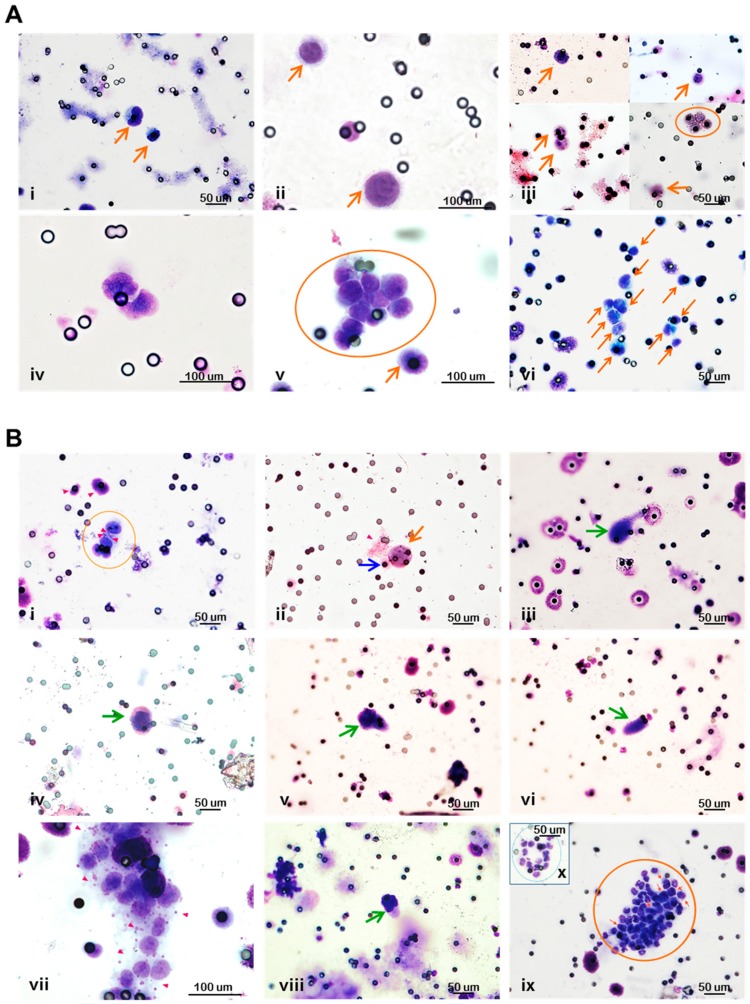
The morphological heterogeneity of CTCs and circulating host cells in BC xenograft models. (**A**) Images are representative of CTC pleomorphism: sCTCs (arrows) with different sizes from (**i**) BT-474 (40× objective) and from (**ii**) MDA-MB-453 (60× oil immersion); (**iii**) sCTCs (arrows) with low (top images) and high (bottom images) nucleus-to-cell ratios, and cCTC (circle) from MDA-MB-468 (40×); (**iv**) the cluster of two CTCs with multilobulated nucleus from MDA-MB-468 (60× oil immersion); (**v**) cCTC from MDA-MB-231 (60× oil immersion); (**vi**) sCTCs and CTCs in clusters (arrows) with different sizes from MDA-MB-231 (40×). (**B**) Images are representative of CTCs and circulating host cells: (**i**) a cluster of four CTCs (circle) and platelets (arrowheads) from BT-474 (40×); (**ii**) sCTC (orange arrow) in cluster with platelets (arrowhead) and one leukocyte (blue arrow) from MDA-MB-453 (40×); (**iii**) atypical giant cells cell from MDA-MB-453 (green arrow, 40×); (**iv**,**v**,**vi**) three atypical giant cells from MDA-MB-468 (green arrow, 40×); (**vii**) cCTC and platelets (arrowheads) from MDA-MB-231 (60× oil immersion); (**viii**) an atypical giant cell from MDA-MB-231 (green arrow, 40×); (**ix**) a cluster (circle) of CTCs (arrows showing clearly distinguishable tumor cells) combined with leukocytes (40×).

Cytological blood samples from different CTC models were analyzed and compared in order to highlight intra-sample and inter-model differences on the basis of morphological criteria (details reported in Materials and Methods). The identified cell subpopulations are hereafter described. As already appreciable in the MDA-MB-231 model (Figure 3, Panel C), single CTCs show a certain degree of morphological heterogeneity (i.e., pleomorphism), each cell with a more or less irregularly outlined nucleus of various sizes and shapes, in addition to the heterogeneity in the whole cell size and morphology (Figure 4, Panel A). The difference in sizes is particularly evident in the two BT-474- and the two MDA-MB-453-derived CTCs depicted in Figure 4, one of them smaller than the other. While in the BT-474 CTC model, the cells display an irregular nucleus, the MDA-MB-453-derived CTCs have a clearly round-shaped nucleus and, besides the larger size, the bigger cell also displays a higher nucleus-to-cell ratio (>0.90) compared to the other, as also indicated by the thinner cytoplasmic rim. sCTCs with low (<0.75) or high (>0.90) nucleus-to-cell ratios were also observed in MDA-MB-468, a few of them also presenting with a multilobulated nucleus. CTCs in the MDA-MB-231 model may present as either round-shaped or polygonal physically interacting cells and may have a widely variable whole size. Interestingly, CTC clusters intermingled with or surrounded by platelets were rarely detected in all models, sporadically also in direct contact with leukocytes, as observed in MDA-MB-453 and MDA-MB-231 (Figure 4, Panel B). Few numbers of cytological figures appearing like atypical giant cells with several shapes (morphological details reported in Materials and Methods), were detected in 13%, 30% and 17% of CTC positive cases from MDA-MB-453, MDA-MB-468 and MDA-MB-231 models, respectively. Despite being present in a minority of CTC +ve cases, atypical giant cells were never found in samples called CTC-negative. Images depicting all cell types described in cytological blood samples from xenograft models is reported in Figure S4. Additionally, a complete list of data describing the presence of circulating cells and metastases in all the analyzed animals for each model is reported in Table S2.

## 4. Discussion

Our study demonstrates the reliability of a technical protocol for CTC and metastasis detection in BC xenograft models based on the classical morphological features of malignancy and relying on the advantage of the species-specificity barrier for the identification of cells of human origin in both cytological blood samples and tissues compared to the host counterpart. The described methodology can be, in principle, applied to every kind of xenograft model for CTC and metastasis biology studies, thanks to its sensitivity and specificity in detecting rare circulating cells and also small metastatic foci in weakly metastatic models. Assessment of metastasis formation using a species-specific antibody was proven a reliable method to identify small and rare metastases, especially in weakly metastatic models.

As such, a preliminary validation test with the metastatic MDA-MB-231 BC cell line showed that hematogenous dissemination and metastases may occur even at the earliest stages upon tumor nodule appearances at the orthotopic site, and can be monitored in a time-course experiment. Here, studies were also performed to model BC metastases using BT-474, MDA-MB-453 and MDA-MB-468 cells, providing a comparative analysis, for the first time, of the hematogenous dissemination potential among BC cell lines belonging to different molecular subtypes, in addition to MDA-MB-231, upon injection at the orthotopic level rather than forced metastasis formation assays. Consistently with the growth rate of the primary tumors, xenografts in the murine model, generated using the HER2 positive BT-474 and MDA-MB-453 and the basal A MDA-MB-468 cell lines, according to Neve et al. [34], determined a lower CTC load compared to the numbers of CTCs generated by MDA-MB-231 xenografts. Interestingly, BC cell line xenografts can generate a pleomorphic CTC population, consisting of markedly heterogeneous cells in terms of the whole cellular and/or nuclear size and morphology, as also nucleus to cell ratio, and which also includes clusters of CTCs, released in blood at a reduced frequency compared to the sCTC subset. Intra-clonal size heterogeneity has been already reported in MDA-MB-231-derived clonal subpopulations in vitro [56], suggesting that upon injection in mice cells with different metastatic abilities and morphological features underwent clonal selection and that such clones might be more easily identifiable in the CTC population in xenografts. However, variable sizes and nuclear-cytoplasmic ratios have been also described in CTCs from prostate cancer patients compared to cultured prostate cancer cell lines [57], again indicating that a clonal selection process takes place at the primary or secondary sites during tumor progression. Therefore, CTC morphological heterogeneity might be interpreted as a hallmark of tumor cells which is likely to be more easily assessable among clones of the blood disseminating population.

We have also demonstrated that not only MDA-MB-231 but also other models may represent experimental tools suitable for CTC characterization and metastasis biology studies. Indeed, each cell line was shown to follow preferential dissemination routes, i.e., through blood or lymphatic vessels, as also distinct colonization patterns at distant sites. Lastly, despite the species-barrier, it was surprising to find, for the first time, circulating cells from the murine host in physical contact with tumor cells of human origin, as also atypical cytological figures.

MDA-MB-231 cells were already proven to induce lung metastases when injected in the tail vein of nude mice [58], and the success of transplantation experiments in the m.f.p. ranked them among the most aggressive BC cell lines [59,60]. Since the first reports, studies employing such a cell line have started to proliferate, and even nowadays they represent a large fraction of the literature on BC metastasis biology. On the contrary, ER-positive cell lines such as MCF7, T47D, and BT-474 are able to form tumors only in the presence of an exogenous source of estrogen. However, despite the metastatic origin of these cell lines, they have a limited ability to invade and metastasize [37], unless subjected to a selection of hormone-resistant variants or genetically modified [61]. More recently, severely immunocompromised mice, such as NSG and Rag2^-/-^ γc^-/-^ models, which exhibit T cell, B cell and natural killer cell immunodeficiency, were also explored to generate new metastatic models. This time, MCF7 were able to give rise to metastases at the lymph-node, lung, spleen and, sporadically, even at the renal level when injected in the m.f.p. of NSG mice [38]. BT-474 cells were instead less metastatic in these mice, generating macro-metastases in only a few cases (axillary lymph node in 17% of mice and the spleen in 8%. of mice). The latter result is in contrast with our model where, although no macrometastases were found, small foci were detected in all animals and in all FFPE sections we have analyzed. In another study, bioimaging analysis enabled the detection of multi-organ metastases in Rag2^-/-^ γc^-/-^ mice injected at the orthotopic level with the MDA-MB-453 and BT-474 cell lines [39].

Attempts to explore hematogenous dissemination in BC experimental models were made only recently. In a technical paper published in 2008 [55], different approaches for blood collection were tested to isolate CTCs from tumor-bearing mice, finally demonstrating that the cardiac puncture represents the most suitable approach to reach high yields without interference from contaminating normal murine epithelial cells. The authors also validated a method to enumerate CTCs by applying a modified version of an in vitro diagnostic system for quantifying CTCs in patients, obtaining numbers of CTCs ranging from ∼100 to 1000 per milliliter of blood. In line with our results, the reported CTC concentration in the blood of MDA-MB-231 xenograft models was highly variable among different animals. Concerning CTC variability, despite the wide range of cells detected in this model, we have observed a correlation between CTC load and tumor burden, whereas the literature data from experiments with GFP-expressing MDA-MB-231 cells suggested that the primary tumor size is not a strong indicator of CTC load [62]. Hence, such a variability in CTC load in experimental models could also be the result of fluctuations in CTC release, as also suggested by results obtained in a melanoma CTC-model [62]. Here, the authors performed real-time continuous monitoring of CTCs and could estimate a release of 0 to 54 CTCs every 5 min, alternated to CTC-free phases. Differently from data obtained in the MDA-MB-231 CTC model [62], this peak in CTC level mirrored the increase in tumor growth at the same time point, suggesting again that the number of CTCs may indeed correlate with tumor size. In our MDA-MB-231 model, probably due to its pronounced aggressiveness, we had no possibility to define an optimal time point to isolate CTCs before they were able to colonize distant organs since in the time-course experiment CTC release and increase during time mirrored the onset, frequency and extent of pulmonary metastases in matched FFPE sections. On the contrary, dissemination via the lymphatic system was observed at the earliest time points (after 35 days from injection), suggesting that different molecular mechanisms are required to enter the lymphatic system compared to blood vessels. In agreement with this observation, Juratli and colleagues [62] also reported that metastatic foci at lymph nodes can be observed just two weeks after orthotopic inoculation. More recently, two seminal works demonstrated that in experimental models, tumor cells invading lymph nodes are able to enter local blood vessels and exit from them to invade distant organs [63,64]. However, commonalities in clinical tumors need to be demonstrated. The results of our dynamic studies with the MDA-MB-231 model also show that CTCs can be actively released from pulmonary metastases. At the same time, once in the bloodstream, MDA-MB-231 cells were able to rapidly reach peripheral districts and colonize the lung as CTC numbers rapidly dropped out upon intravenous injection and their presence was generally associated to the presence of lymph-node and lung metastases, even at early time points. Different results were observed in a MDA-MB-468 CTC-model already described by Bonnomet and colleagues [65], where CTCs were detectable as early as 8 days after injection and increased 36 days later, after which their levels remained quite constant. Indeed, Bonnomet and colleagues also found lung metastases at later time point only, despite CTC recovery was possible even a few days after cell injection in a time-course experiment [65].

An interesting result emerging from our study is the detection of clusters of CTCs in all the examined BC models. Overall, to our knowledge, the presence of cCTCs has been reported for the triple-negative LM2 MDA-MB-231 [66] and MDA-MB-435 [67] BC cell lines only. We were able to monitor the presence of CTCs using size-based isolation support also in xenograft models obtained from BC cells with different molecular subtypes. Consistently with the demonstration that cCTCs are endowed with higher efficiency in initiating metastasis [66,68], the frequency of cCTCs was 2-fold lower in models with weak metastatic potential compared to MDA-MB-231. Moreover, CTCs generated upon xenograft of BC cell lines were heterogeneous in morphology, even within the same cluster, thus suggesting further heterogeneity at the molecular level, probably as a result of a selection process of cells which are committed to disseminate in blood according to their functionality and ability to cooperate and survive in a foreign microenvironment. Unexpectedly, some clusters of tumor cells also presented with physically interacting platelets and/or leukocytes. CTCs in contact with blood or stromal cells were already described both in experimental models and clinical samples and some cell types, such as platelets, neutrophils and monocytes/macrophages, were also demonstrated to promote epithelial-to-mesenchymal transition and the pre-metastatic niche formation [69,70,71,72], even at early BC stages [73], or to assist CTCs during their transit in blood and organ colonization, increasing their metastatic ability [74]. However, to our knowledge, interactions between circulating tumor and host cells in xenografts have not been reported at present. Platelet depletion in a nude mouse model transplanted subcutaneously with SKOV3 ovarian cancer cells provided evidence that platelets are involved in cancer cell growth [75], hence suggesting that even cells from the murine compartment can be actively recruited to cooperate with human tumor cells in xenograft models. With reference to our data, the growth rate of matched axillary and inguinal m.f.p. nodules generally showed a different trend in both MDA-MB-468 and MDA-MB-231 models, with a higher tumor mass at the axillary compared to the inguinal site, even statistically significant during the latest time point measurements. Such data are consistent with the hypothesis that, despite the species specificity barrier, tumor cells can interact and possibly cross-talk with the murine microenvironment in xenograft models. Finally, atypical giant cells, presenting without features of malignancy and, therefore, expected to originate from the host, were also observed in our models. Similarly to clinical samples [52], such a cell type has been generally associated with the presence of CTCs and never found in CTC-negative samples. The origin of circulating atypical giant cells in xenografts has not been investigated here. Our hypothesis is that cells displaying not all the classical features of malignancy and presenting with unusual morphological patterns, which resemble those described for cancer-associated macrophage-like cells by Adams and colleagues [52], might derive from the interaction between tumor and host cells. Fusion hybrids, i.e., hybrid cells derived from fusion events between tumor cells and macrophages, were already described in murine experimental models [76]. If such cells originate in response to the attack from the immune system towards tumor cells, or as a strategy to increase the tumor cell viability and metastatic potential, is yet to be established. Literature data reported that murine peritoneal macrophages can phagocytize apoptotic BC tumor cells from cell lines in vitro, and acquire stem-like features in the following steps [77]. With reference to the xenograft milieu, another study reported the host macrophage invasion and the presence of multinuclear giant cells or foreign body giant cells at the implant site upon the injection of human mesenchymal stem cells with biopolymers in NOD scid mice, thus indicating that severely immunocompromised mice are able to retain a certain level of innate immune responsiveness [78]. On the other hand, cell fusion has been associated with the acquisition of increased metastatic capacity or enhanced drug resistance [79], and the presence of circulating hybrid cells was shown to correlate with the disease stage and patient survival [76]. Overall, we are aware that beside the identification based on morphological criteria although performed by a pathologist experienced in CTC detection in clinical samples, only a molecular characterization of such atypical populations of cells, including leukocytes interacting with CTCs, e.g., with species-specific antibodies and gene expression analyses, would definitely elucidate their nature and confirm the validity of xenograft models for new research lines.

## 5. Conclusions

In the end, CTCs and metastases can be in vivo modeled from BC cell lines with different subtypes and disseminating potential. In xenografts, several subpopulations of cells circulating in the blood can be identified by applying the classical morphological criteria, thus offering experimental models alternative to MDA-MB-231, as unusual and intriguing tools to investigate tumor-host interactions in the blood microenvironment.

## Figures and Tables

**Table 1:** Scheme of breast cancer cell line xenotransplantation in immunocompromised mice.

**Cell Line**	**Mouse Model**	**Number of Cells per Injection**	**Injection Sites (m.f.p.)**
BT-474	NSG	5 × 10^6^	4th left
MDA-MB-453	NSG	10^7^	4th left
MDA-MB-468	NSG	5 × 10^6^	2nd right and 4th left
MDA-MB-231	NOD scid	5 × 10^6^	2nd right and 4th left

**Table 2:** The metastasis sites and frequencies in breast cancer xenograft models.

CTC-Model	Experiment 1									Experiment 2								
	N	Lymph-Node		Lungs		Ovary 1		Ovary 2		N	Lymph-Node		Lungs		Ovary 1		Ovary 2	
		+ve Cases	Positivity Frequency (%) *	+ve Cases	Positivity Frequency (%) *	+ve Cases	Positivity Frequency (%) *	+ve Cases	Positivity Frequency (%) *		+ve Cases	Positivity Frequency (%) *	+ve Cases	Positivity frequency (%) *	+ve Cases	Positivity Frequency (%) *	+ve Cases	Positivity Frequency (%) *
BT-474	3	- ^§^	-	3	25–100 ^†^	0	-	0	-	7	- ^§^	-	7	100	0	-	0	-
MDA-MB-453	3	- ^§^	-	2	80–100 ^†^	0	-	0	-	7	- ^§^	-	7	75–100 ^†^	6	75–100 ^†^	5 ^‡^	43–100 ^†^
MDA-MB-468	3	- ^§^	-	3	100	1	21	0	-	7	7	100	7	100	2	23–100 ^†^	1	90
MDA-MB-231	5	5	100	5	100	- ^¥^	-	- ^¥^	-	6	6	100	6	100	- ^¥^	-	- ^¥^	-

* range of positivity frequencies (number of metastasis-positive (+ve) out of 10 to 24 sections in Experiment 1, and out of 24 to 48 sections in Experiment 2). § not detectable or not involved at the macroscopic level. ‡ 1 out of 2 ovaries not assessable in one case. † positivity range. ¥ not assessed.

## Supplementary Materials

The following are available online at https://www.mdpi.com/2073-4409/8/7/683/s1, Figure S1: Species-specificity of anti-human COX IV antibody; Figure S2: Primary tumor growth in breast cancer xenograft models; Figure S3: Lung metastases in breast cancer xenograft models; Figure S4: Cell types observed in cytological blood samples from breast cancer xenograft models; Table S1: Circulating tumor cell load in breast cancer xenograft models; Table S2: Circulating cells and metastases in xenograft models from breast cancer cell lines.
