# Supplementary material for: The Detection and Morphological Analysis of Circulating Tumor and Host Cells in Breast Cancer Xenograft Models
Source: Cells. 2019 Jul 5;8(7):683. doi: 10.3390/cells8070683 (PMC6679018; doi:10.3390/cells8070683)
Supplement: Supplementary file 1 [file cells-08-00683-s001.pdf]

SUPPLEMENTARY MATERIALS

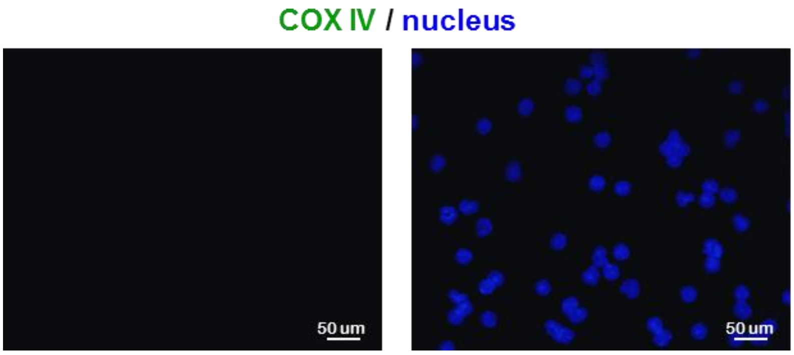

Figure S1. Species-specificity of anti-human COX IV antibody. Image represents spleen mononuclear cells from a BALB/c Nude mouse acquired by FITC (anti-COX IV antibody, *left*) and DAPI (nuclei, *right*) filters (40x oil immersion objective).

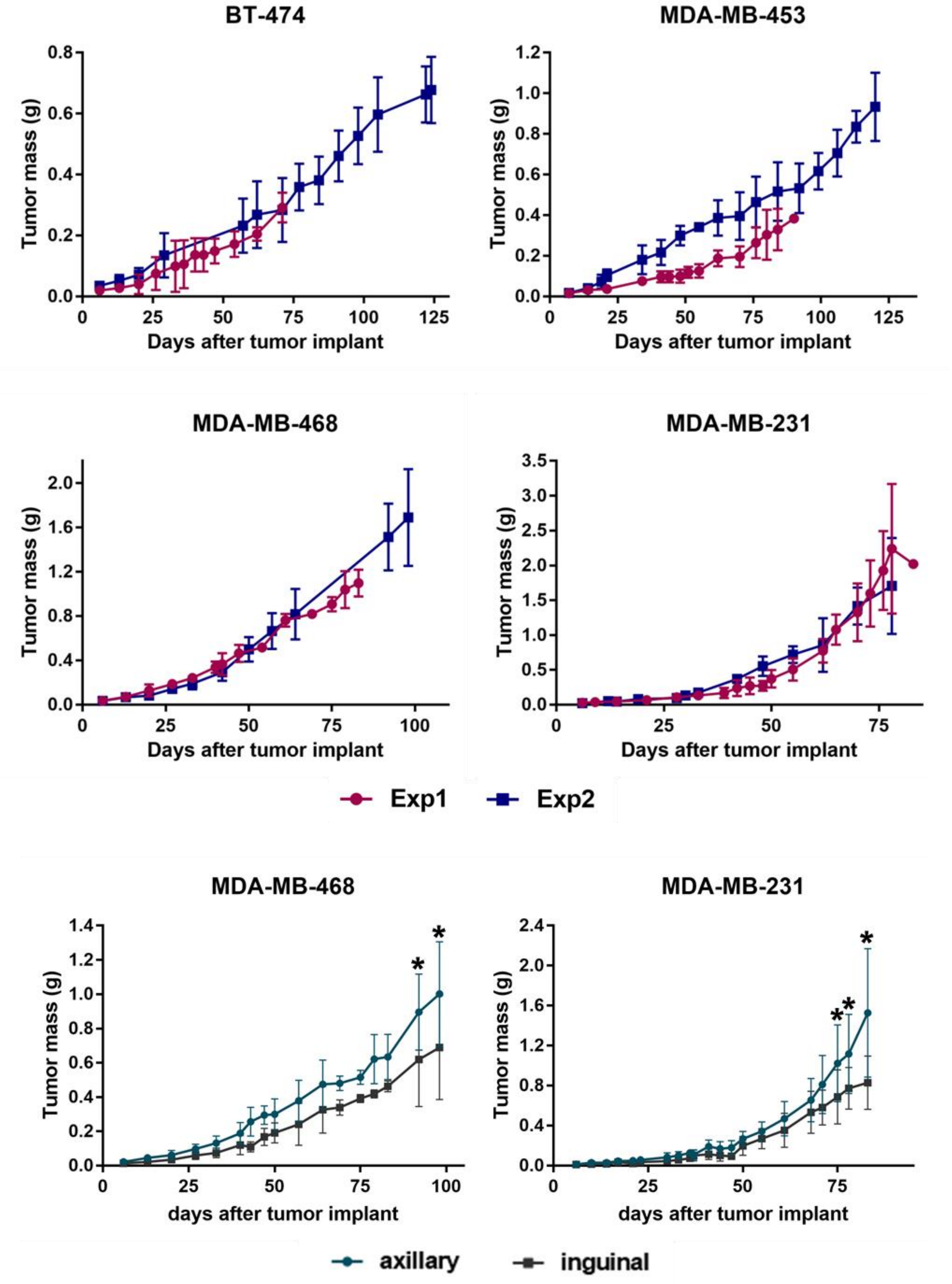

Figure S2. Primary tumor growth in breast cancer xenograft models. Curves represent the mean±SD mass of a single nodule for BT-474 and MDA-MB-453, the mean±SD of the sum of the masses of two nodules for

MDA-MB-468 and MDA-MB-231, and the mean±SD masses of matched axillary and inguinal nodules from MDA-MB-468 and MDA-MB-231 xenograft models, at different time points (“*”: adjusted p-value <0.01).

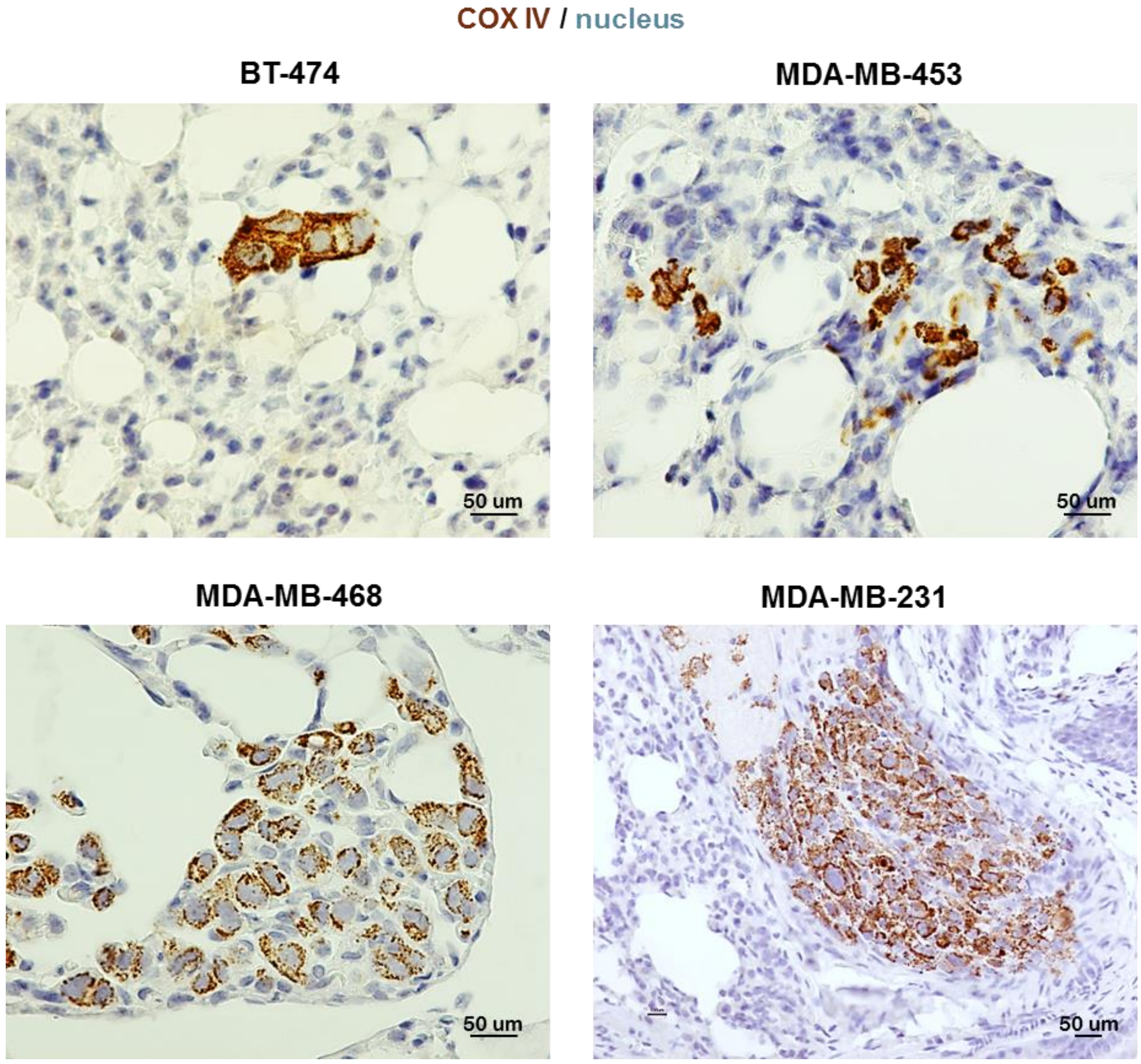

Figure S3. Lung metastases in breast cancer xenograft models. Images represent metastatic cell foci, with tumor cells showing cytoplasmic punctuated brown staining for COX IV and blue counterstained large nuclei, in lung samples from BT-474, MDA-MB-453, MDA-MB-468 (40x oil immersion objective) and MDA-MB-231 (20x objective) xenograft models.

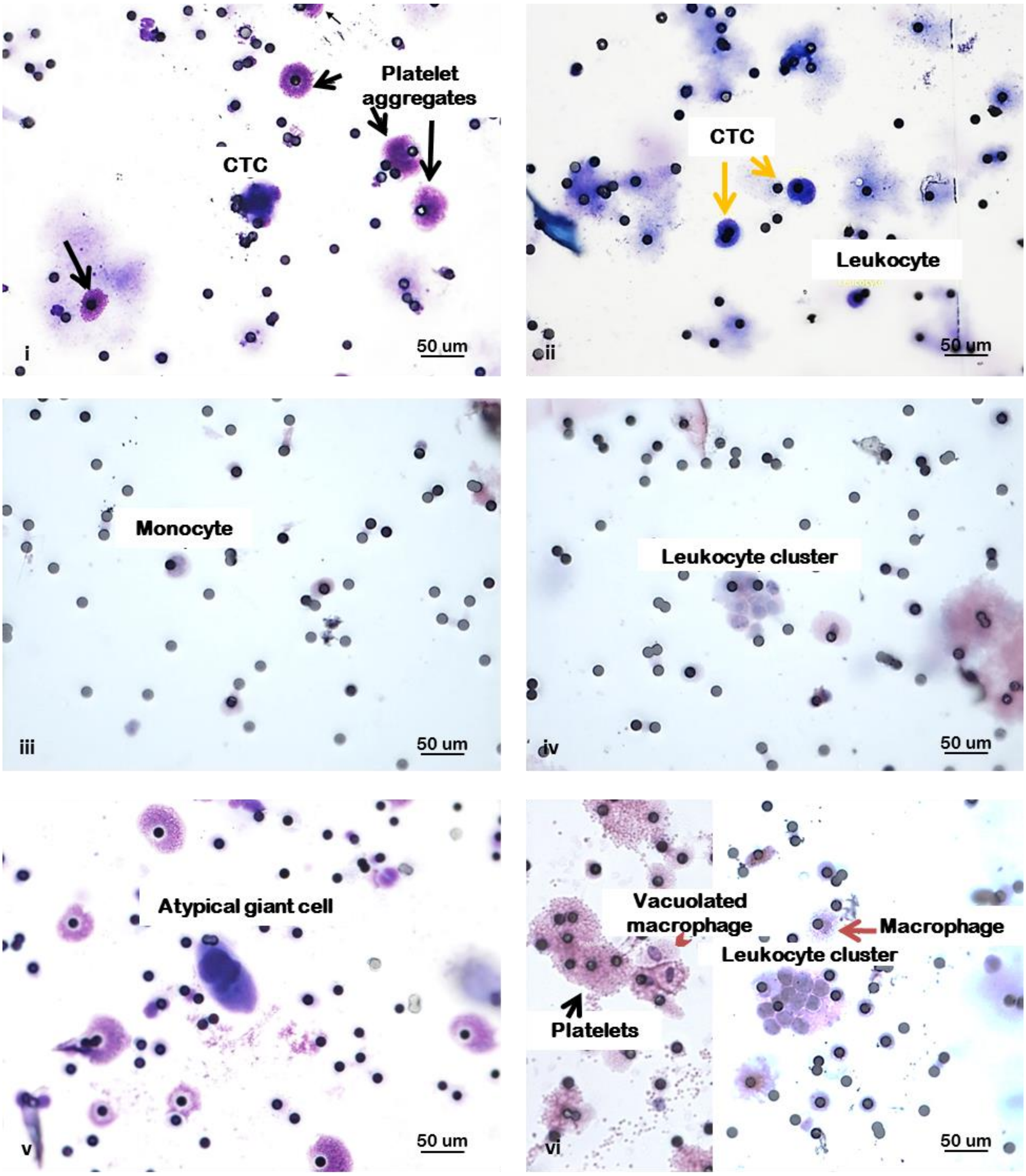

Figure S4. Cell types observed in cytological blood samples from breast cancer xenograft models. Images depict (**i**) platelet aggregates (black arrows) in MDA-MB-231 xenografts (40 objective), (**ii**) a single leukocyte, (**iii**) a monocyte, (**iv**) a cluster of leukocytes and (**v**) an atypical giant cell in MDA-MB-453 xenografts (40x objective), and (**vi**) a vacuolated macrophage in MDA-MB-453 (*left*) and a normal macrophage (*right*) in BT-474 xenografts (red arrows, 40x objective).

Table S1. Circulating tumor cell (CTC) load in breast cancer xenograft models

CTC model	median*(range) sCTC/mL				median*(range) cCTC/mL				overall mean±SD	
	Exp1		Exp2		Exp1		Exp2			
	N**	sCTC	N*	sCTC	N*	cCTC	N**	cCTC	sCTC/mL	cCTC/mL
**BT-474**	2	0-2	7	1(1-5)	2	0-2	6	0(0-2)	1.8±1.6	0.5±0.9
**MDA-MB-453**	3	0-149	5	6(1-800)	3	0-1	5	0(0-1)	122.3±278.5	0.5±0.5
**MDA-MB-468**	3	2-5	7	2(0-8)	3	0-4	7	0(0-2)	3.5±2.6	1.1±1.5
**MDA-MB-231**	5	7(0-9,625)	6	2(0-417)	6	16(1-5,973)	6	1(0-10)	946.9±2,882.0	560.2±1,796.0

ABBREVIATIONS: sCTC: single circulating tumor cell; cCTC: CTC cluster; SD: standard deviation; Exp: experiment

* if N≥5

**number of cases assessable for sCTC or cCTC count according to sample quality

Table S2. Circulating cells and metastases in xenograft models from breast cancer cell lines.

Cell line	Exp n°	Sacrifice day	sCTC	cCTC	AGC	Other atypical cells and clusters	Pulmonary metastases*	Lymph-nodal metastases*	Ovarian metastases*
BT-474	Exp 1	71	pos	pos	neg	.	NA	NA	NA
BT-474	Exp 1	75	neg	neg	neg	.	NA	NA	NA
BT-474	Exp 1	81	NE	NE	NE	.	NA	NA	NA
BT-474	Exp 2	91	pos	neg	neg	.	pos	neg	neg
BT-474	Exp 2	91	pos	neg	neg	.	pos	neg	neg
BT-474	Exp 2	105	pos	NE	NE	.	pos	neg	neg
BT-474	Exp 2	124	pos	pos	neg	1 CTCs-platelets cluster	pos	neg	neg
BT-474	Exp 2	124	pos	neg	neg	.	pos	neg	neg
BT-474	Exp 2	105	pos	neg	neg	.	pos	neg	neg
BT-474	Exp 2	124	pos	neg	neg	.	pos	neg	neg
MDA-MB-453	Exp 1	86	pos	neg	neg	vacuolated macrophages; 1 CTC-platelet-leukocyte cluster; 1 mitotic figure (CTC)	neg	NA	neg
MDA-MB-453	Exp 1	90	neg	pos	neg	.	pos	NA	neg
MDA-MB-453	Exp 1	90	pos	pos	neg	.	pos	NA	neg
MDA-MB-453	Exp 2	85	pos	neg	neg	.	pos	neg	pos
MDA-MB-453	Exp 2	119	pos	pos	neg	.	pos	neg	pos
MDA-MB-453	Exp 2	119	pos	pos	pos	.	pos	neg	pos
MDA-MB-453	Exp 2	119	pos	neg	neg	.	pos	neg	pos
MDA-MB-453	Exp 2	103	NA	NA	NE	.	NA	NA	NA
MDA-MB-453	Exp 2	103	NE	NE	NE	.	pos	neg	pos
MDA-MB-453	Exp 2	103	pos	neg	neg	2 mitotic figures (CTCs)	pos	neg	pos
MDA-MB-468	Exp 1	85	pos	pos	pos	1 out of 4 CTCs-platelets cluster	NA	neg	NA
MDA-MB-468	Exp 1	85	pos	pos	neg	.	NA	neg	NA
MDA-MB-468	Exp 1	85	pos	neg	neg	.	NA	neg	NA
MDA-MB-468	Exp 2	92	neg	neg	neg	.	pos	pos	pos
MDA-MB-468	Exp 2	92	pos	pos	pos	.	pos	pos	neg
MDA-MB-468	Exp 2	98	pos	neg	pos	.	pos	pos	neg
MDA-MB-468	Exp 2	98	pos	neg	neg	.	pos	pos	neg

Table S2. ***continued***

MDA-MB-468	Exp 2	98	pos	neg	neg	.	pos	pos	neg
MDA-MB-468	Exp 2	98	pos	pos	neg	1 CTCs-platelets cluster	pos	pos	neg
MDA-MB-468	Exp 2	98	pos	neg	neg	.	pos	pos	pos
MDA-MB-231	Exp 1	28	NE	NE	NE	.	neg	neg	NA
MDA-MB-231	Exp 1	35	neg	NE	NE	.	neg	neg	NA
MDA-MB-231	Exp 1	35	pos	pos	neg	.	pos	neg	NA
MDA-MB-231	Exp 1	35	neg	neg	neg	.	neg	pos	NA
MDA-MB-231	Exp 1	36	neg	neg	neg	.	neg	pos	NA
MDA-MB-231	Exp 1	35	neg	NE	neg	.	neg	pos	NA
MDA-MB-231	Exp 1	50	pos	pos	neg	.	neg	neg	NA
MDA-MB-231	Exp 1	50	neg	pos	neg	Numerous CTCs mixed with leukocytes	pos	neg	NA
MDA-MB-231	Exp 1	50	neg	neg	neg	.	neg	pos	NA
MDA-MB-231	Exp 1	50	NE	NE	NE	.	pos	pos	NA
MDA-MB-231	Exp 1	50	NE	NE	NE	.	pos	pos	NA
MDA-MB-231	Exp 1	49	NE	pos	pos	.	neg	pos	NA
MDA-MB-231	Exp 1	62	pos	pos	NE	1 CTCs-leukocytes cluster; 3 CTCs-platelets clusters; presence of apoptotic CTCs	pos	pos	NA
MDA-MB-231	Exp 1	62	neg	pos	pos	.	pos	neg	NA
MDA-MB-231	Exp 1	65	neg	pos	neg	.	pos	pos	NA
MDA-MB-231	Exp 1	65	pos	pos	neg	.	pos	pos	NA
MDA-MB-231	Exp 1	65	neg	pos	pos	2 apoptotic CTCs	neg	neg	NA
MDA-MB-231	Exp 1	65	pos	pos	pos	2 apoptotic CTCs	pos	pos	NA
MDA-MB-231	Exp 1	83	pos	pos	neg	.	pos	pos	NA
MDA-MB-231	Exp 1	84	neg	pos	neg	.	pos	pos	NA
MDA-MB-231	Exp 1	83	pos	pos	pos	1 CTCs-leukocytes cluster	pos	pos	NA
MDA-MB-231	Exp 1	83	pos	pos	neg	.	pos	neg	NA
MDA-MB-231	Exp 1	84	pos	pos	neg	.	pos	pos	NA
MDA-MB-231	Exp 2	77	pos	pos	neg	.	pos	pos	NA
MDA-MB-231	Exp 2	77	pos	pos	neg	.	pos	pos	NA
MDA-MB-231	Exp 2	77	pos	neg	neg	.	pos	pos	NA

Table S2. ***continued***

MDA-MB-231	Exp 2	79	neg	neg	neg	.	pos	pos	NA
MDA-MB-231	Exp 2	79	pos	neg	neg	.	pos	pos	NA
MDA-MB-231	Exp 2	77	pos	pos	neg	4 CTCs-platelets clusters	pos	pos	NA

ABBREVIATIONS: CTC: circulating tumor cell; sCTC: single CTC; cCTC: CTC cluster; AGC: atypical giant cell; NA: not available; NE: not evaluable; Exp: experiment

* data refers to results of immunohistochemistry analysis
